# Inducible RNA targeting and *N*^6^-methyladenosine editing by a split-Cas13 architecture

**DOI:** 10.1093/jmcb/mjae002

**Published:** 2024-01-12

**Authors:** Yang Li, Qiang Sun, Zhi Yang, Gang Yuan

**Affiliations:** International Institutes of Medicine, the Fourth Affiliated Hospital of Zhejiang University School of Medicine, Yiwu 322000, China; International Institutes of Medicine, the Fourth Affiliated Hospital of Zhejiang University School of Medicine, Yiwu 322000, China; Future Health Laboratory, Innovation Center of Yangtze River Delta, Zhejiang University, Jiaxing 314100, China; Department of Thoracic Surgery and Institute of Thoracic Oncology, West China Hospital, Sichuan University, Chengdu 610041, China; Department of Thoracic Surgery and Institute of Thoracic Oncology, West China Hospital, Sichuan University, Chengdu 610041, China

**Keywords:** split-Cas13, mRNA, m^6^A, CRISPR–CasRx, chemically induced dimerization

**Dear editor**,

Cas13, a class II type VI CRISPR‒Cas endonuclease with two higher eukaryotes and prokaryotes nucleotide-binding (HEPN) domains, exclusively binds to and cleaves single-stranded RNA by a complementary guide RNA (gRNA) (Makarova et al., 2020). RfxCas13d (also known as CasRx) is an ortholog of CRISPR‒Cas13 with a relatively small size and robust RNA knockdown capability in mammalian cells (Konermann et al., 2018). Owing to its simplicity and high efficiency, CasRx has been applied for RNA targeting in various types of cells and organisms. Nevertheless, most of these studies failed to control the nuclease activity of CasRx spatiotemporally. The re-engineered split-Cas9 system enables inducible genome editing and epigenetic modulation through chemical-induced reassembly of the Cas9 protein (Zetsche et al., 2015). However, tools that allow for efficient and controllable RNA knockdown by a split-Cas13 system remain to be established.

In this study, we aimed to develop a split-Cas13 system that enables inducible RNA knockdown with a precise spatiotemporal control of specific transcripts. For the split-Cas9 system, previous studies utilized two different schemes: (i) the Cas9 protein was directly split into N-terminal and C-terminal fragments (Zetsche et al., 2015), and (ii) the Cas9 protein was split into recognition and nuclease lobes (Wright et al., 2015). As a result, the split-Cas9 system based on the first scheme has higher genome editing efficiency. Therefore, we applied the first scheme to design the split-Cas13 architecture, in which the Cas13 protein was split into an N-terminal fragment Cas13(N) and a C-terminal fragment Cas13(C). The chemically induced dimerization (CID) system has been applied to modulate various cellular processes through small molecule-induced protein proximity (Voss et al., 2015). Among all CID systems, rapamycin-induced FK506 binding protein (FKBP) and FKBP–rapamycin binding (FRB) dimerization are widely utilized (Banaszynski et al., 2005; Zetsche et al., 2015). Thus, we proposed that the separated Cas13(N)-FRB and FKBP-Cas13(C) fusion proteins could be reassembled into intact and functional Cas13 protein upon rapamycin induction to conduct RNA cleavage and degradation (Figure 1A).

**Figure 1:**
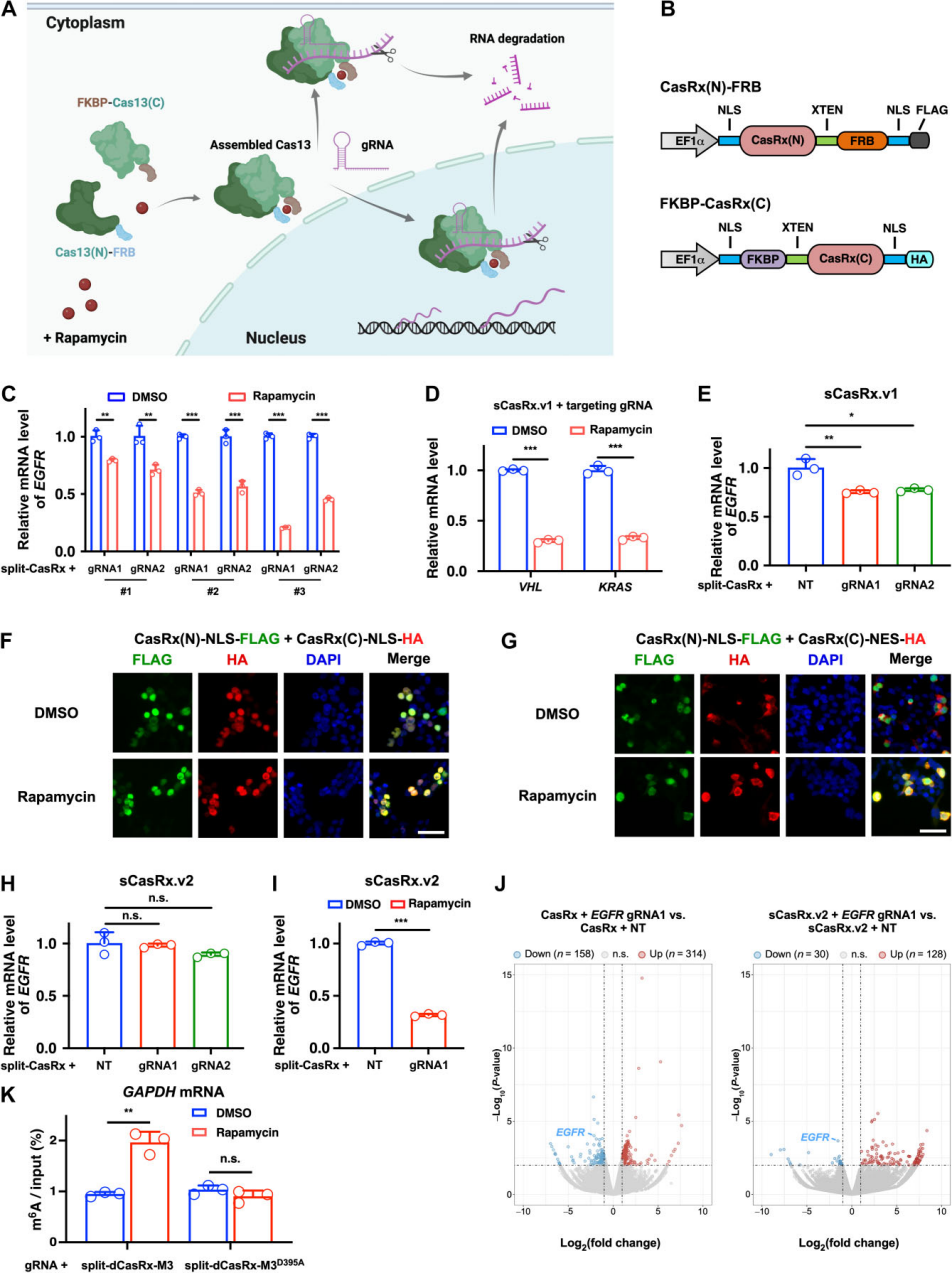
Inducible RNA knockdown and m^6^A editing by a split-CasRx architecture. (**A**) Schematic illustration of the rapamycin-inducible split-Cas13 system. (**B**) Construction of NLS-CasRx(N)-FRB-NLS and NLS-FKBP-CasRx(C)-NLS fusion proteins (sCasRx.v1). (**C**) sCasRx.v1-mediated knockdown of *EGFR* mRNA in the presence of rapamycin. (**D**) sCasRx.v1-mediated knockdown of *VHL* and *KRAS* mRNAs in the presence of rapamycin. (**E**) sCasRx.v1-mediated knockdown of *EGFR* mRNA in the absence of rapamycin. (**F**) Cellular localization of the CasRx(N) and CasRx(C) fragments of sCasRx.v1. Scale bar, 50 μm. (**G**) Cellular localization of the CasRx(N) and CasRx(C) fragments of the optimized sCasRx.v2. Scale bar, 50 μm. (**H**) sCasRx.v2-mediated knockdown of *EGFR* mRNA in the absence of rapamycin. Compared with sCasRx.v1, sCasRx.v2 mediated less background RNA knockdown. (**I**) sCasRx.v2-mediated knockdown of *EGFR* mRNA in the presence of rapamycin. (**J**) Volcano plots showing that compared with intact CasRx, sCasRx.v2-mediated RNA knockdown affected less non-targeted mRNAs. Two biological replicates were performed. (**K**) Validation of rapamycin-induced RNA m^6^A deposition by split-dCasRx and M3 conjugates. NT, non-targeting gRNA. Values and error bars represent mean ± standard deviation. n.s., not significant; *P < 0.05; **P < 0.01; ***P < 0.001.

First, a split-CasRx system was constructed. By combining AlphaFold2-predicted protein structure and domain organization of the CasRx protein, three split sites located in both the structured and unstructured regions were identified (Supplementary Figure S1). Because both HEPN domains contain catalytic residues and are indispensable for gRNA-dependent RNA cleavage of the Cas13 protein (Konermann et al., 2018; Zhang et al., 2018), we separated the two HEPN domains into CasRx(N) and CasRx(C), respectively. Then, CasRx(N) and CasRx(C) were respectively fused to the FRB and FKBP domains using a 16-residue XTEN linker. Because nucleus-localized CasRx shows high RNA knockdown efficiency in human cells (Konermann et al., 2018), we also fused the nuclear localization signal (NLS) to both termini of CasRx(N)-FRB and FKBP-CasRx(C) (Figure 1B). In addition, a FLAG or HA tag was introduced to the C-terminus of the CasRx(N)-FRB or FKBP-CasRx(C) construct, respectively (Figure 1B).

Next, we investigated the effectiveness of the split-CasRx system in targeting endogenous mRNAs. Two gRNAs targeting *EGFR* mRNA were designed and could significantly reduce endogenous *EGFR* mRNA level when co-transfected with CasRx in HEK293T cells (Supplementary Figure S2A). With the induction of 200 nM rapamycin, each of the three split-CasRx constructs co-transfected with the complementary gRNAs resulted in a significant decrease in *EGFR* mRNA level, with split-CasRx#3 showing the highest knockdown efficiency (Figure 1C). Thus, the combination of the NLS-CasRx(N)#3-XTEN-FRB-NLS and NLS-FKBP-XTEN-CasRx(C)#3-NLS constructs was designated as the first-generation inducible split-CasRx system (sCasRx.v1). Comparable to intact CasRx, sCasRx.v1 co-transfected with effective gRNAs also significantly suppressed *VHL* and *KRAS* mRNA levels in response to rapamycin induction (Figure 1D; Supplementary Figure S2B), confirming that sCasRx.v1-mediated RNA knockdown is broadly applicable.

Furthermore, we designed a split-PspCas13b system based on structural prediction by AlphaFold2 and domain organization of the PspCas13b protein (Supplementary Figure S3). With the induction of 200 nM rapamycin, co-expression of split-PspCas13b#1 or #2 and the complementary gRNAs resulted in a modest reduction in *PNPLA2* mRNA level (Supplementary  Figure S4A and B). The combination of split-PspCas13b#2 and corresponding gRNAs also significantly suppressed *VHL* and *KRAS* mRNA levels in a rapamycin-inducible manner (Supplementary  Figure S4C and D). Moreover, transcriptome-wide differential gene expression analysis did not show any obvious alteration in the global transcriptome upon rapamycin induction (Supplementary  Figure S5A). Real-time quantitative polymerase chain reaction further verified that the expression levels of all the target mRNAs (*EGFR, PNPLA2, VHL*, and *KRAS*) were not affected by rapamycin treatment (Supplementary  Figure S5B), ruling out the possibility that the alteration in mRNA level was due to rapamycin induction itself. Taken together, these findings demonstrate that both CasRx and PspCas13b can be re-engineered into a rapamycin-inducible split-Cas13 system, which enables efficient RNA knockdown in human cells.

A previous study showed that the Cas9 split fragments could assemble spontaneously without chemical induction (Zetsche et al., 2015). To explore whether this feature also exists in the split-Cas13 system, we examined the RNA knockdown efficiency of sCasRx.v1 without rapamycin induction. Indeed, co-expression of sCasRx.v1 and the complementary gRNAs in the absence of rapamycin resulted in a mild but significant decrease in *EGFR* mRNA level (Figure 1E). In addition, immunofluorescence staining of HEK293T cells transfected with the sCasRx.v1 constructs revealed that both CasRx(N) and CasRx(C) fragments were located primarily in the cell nucleus, irrespective of the absence or presence of rapamycin (Figure 1F). These results indicate that the CasRx split fragments can undergo auto-assembly and thus hinder the precise control of target mRNAs when using sCasRx.v1. We hypothesized that confining the CasRx(N) and CasRx(C) fragments to different cellular compartments might reduce or eliminate the background RNA knockdown mediated by sCasRx.v1. To confine the FKBP-CasRx(C) construct to the cytoplasm, the N-terminal NLS was removed, and the C-terminal NLS was replaced with a nuclear export signal (NES) (Supplementary  Figure S6A). We speculated that under normal conditions, CasRx(N)-FRB and FKBP-CasRx(C) are spatially confined to the nucleus and cytoplasm, respectively, while upon rapamycin induction, NLS-CasRx(N)-FRB-NLS dimerizes with FKBP-CasRx(C)-NES in the cytoplasm and the assembled CasRx translocates into the nucleus to conduct RNA binding and cleavage (Supplementary  Figure S6B). Here, we designated the combination of NLS-CasRx(N)#3-XTEN-FRB-NLS and FKBP-XTEN-CasRx(C)#3-NES constructs as the second-generation inducible split-CasRx system (sCasRx.v2). As expected, in HEK293T cells transfected with the sCasRx.v2 constructs in the absence of rapamycin, CasRx(N) was confined to the cell nucleus, while CasRx(C) was located mainly in the cytoplasm (Figure 1G). Upon rapamycin induction, CasRx(N) and CasRx(C) fragments dimerized and co-localized in the nucleus (Figure 1G). Consequently, the background RNA knockdown mediated by sCasRx.v2 in the absence of rapamycin was negligible (Figure 1H), while co-expression of sCasRx.v2 and the complementary gRNA in the presence of rapamycin resulted in efficient *EGFR* mRNA knockdown (Figure 1I). In addition, co-transfection of sCasRx.v2 with effective gRNAs that target *EGFR, VHL*, and *GAPDH* mRNAs resulted in simultaneous knockdown of all three mRNAs with the induction of rapamycin (Supplementary  Figure S7). These results indicate that the optimized sCasRx.v2 with significantly reduced background RNA knockdown can be applied for precise RNA knockdown upon rapamycin induction.

Although the CRISPR–CasRx system has been recognized as a promising platform for efficient RNA targeting, its off-target effects in mammalian cells still pose challenges for its *in vitro* and *in vivo* applications. To examine whether sCasRx.v2, which enables the spatiotemporal control of RNA cleavage, can reduce the off-target effects, we performed transcriptome-wide RNA-sequencing (RNA-seq) analysis. Both sCasRx.v2 and intact CasRx were capable of silencing endogenous *EGFR* mRNA when complexed with the complementary gRNA, but sCasRx.v2-mediated RNA knockdown resulted in much less differentially expressed non-targeted mRNAs compared with intact CasRx (Figure 1J). These findings indicate that sCasRx.v2 not only enables efficient RNA knockdown but also markedly alleviates the off-target effects of CasRx.

Finally, we explored whether the split-CasRx architecture could be applied to catalytically dead CasRx (dCasRx) for inducible editing of RNA *N*^6^-methyladenosine (m^6^A) modification. Since m^6^A deposition often happens co-transcriptionally, we speculated that the split-dCasRx architecture tethered with the RNA methyltransferase domain of METTL3 (referred to as M3 hereafter) (Wang et al., 2016) can localize to the nucelus and mediate m^6^A installation at target sites in a gRNA-dependent and rapamycin-inducible manner. To establish the split-dCasRx system, the catalytic residues in the HEPN domains were mutated to alanine (R239A/H244A/R858A/H863A), and M3 was fused to the C-terminus of the dCasRx(C) fragment (Supplementary  Figure S8). As a result, the conjugation of split-dCasRx with wild-type M3, rather than the catalytically impaired M3^D395A^ mutant, mediated successful m^6^A deposition on the lowly methylated A690 site of *GAPDH* mRNA when supplied with the complementary gRNA and rapamycin (Figure 1K), validating rapamycin-induced RNA m^6^A deposition by the split-dCasRx system.

In summary, the split-Cas13 architecture allows for precise spatiotemporal control of target RNA levels in human cells. Our findings suggest that the split fragments are more likely to reassemble into a functional CasRx protein when the split site is located in unstructured regions. Moreover, both the CasRx(N) and CasRx(C) fragments have significantly smaller molecular sizes than the intact CasRx protein, allowing for the incorporation of additional regulatory elements, such as cell-specific promoters. This feature could facilitate adeno-associated virus-based delivery of the CRISPR–CasRx-related system and provide a new platform for exquisitely controlling the expression levels of target transcripts in specific cell types *in vivo*. Recently, two other groups also developed similar split-Cas13 platforms based on different CID systems, which enable inducible RNA knockdown (Ding et al., 2023; Xu et al., 2023). Thus, further research is needed to explore the best application scenarios of different split-Cas13 systems, because their CID systems, RNA silencing efficiencies, and off-target effects vary from one another.

*[Supplementary material is available at Journal of Molecular Cell Biology online. This work was supported by the National Natural Science Foundation of China (82203444 and 82273161) and the Natural Science Foundation of Jiangsu Province (BK20220672). Y.L. and G.Y. conceived the project and designed the experiments. Y.L., Q.S., and Z.Y. performed the experiments and analyzed the data. Q.S. analyzed the RNA-seq data. Y.L. and Q.S. wrote the manuscript with the help of the other authors. Y.L. and G.Y. co-supervised the project.]*

## Supplementary Material

mjae002_Supplemental_File
