# Supplementary material for: Inducible RNA targeting and N6-methyladenosine editing by a split-Cas13 architecture
Source: J Mol Cell Biol. 2024 Jan 12;16(1):mjae002. doi: 10.1093/jmcb/mjae002 (PMC11262032; doi:10.1093/jmcb/mjae002)
Supplement: mjae002_Supplemental_File [file mjae002_supplemental_file.pdf]

Supplementary materials

Table of Contents

Experiment Procedures 2

References 4

Supplementary results

Supplementary Figure S1. The predicted structure and split sites of the CasRx protein 5

Supplementary Figure S2. Inducible RNA knockdown by sCasRx.v1 6

Supplementary Figure S3. The predicted structure and split sites of the PspCas13b protein 7

Supplementary Figure S4. Validation of the inducible split-Cas13b system 8

Supplementary Figure S5. Rapamycin treatment shows no obvious effect on target RNAs 9

Supplementary Figure S6. Optimization of the split-CasRx system 10

Supplementary Figure S7. Inducible knockdown of multiple mRNAs by sCasRx.v2 11

Supplementary Figure S8. Construction of the inducible RNA m^6A editing tool 12

Supplementary Table S1. Primers used in this study 13

Supplementary Table S2. Guide RNA spacer sequences used in this study 14

Supplementary Sequences 1. The amino acid sequences of sCasRx.v1 fusions 15

Supplementary Sequences 2. The amino acid sequences of sCasRx.v2 fusions 16

Supplementary Sequences 3. The amino acid sequences of split-dCasRx-M3/M3^{D395A} fusions 17

Supplementary materials

Experimental Procedures

Plasmid Constructions

To generate split-CasRx and split-PspCas13b constructs, split fragments of CasRx, dCasRx, and PspCas13b were cloned with indicated primers (Table S1) using Phanta Max Super-Fidelity DNA Polymerase (Vazyme). The vector pXR001-EF1a-CasRx (Addgene plasmid no. 109049) was also linearized with the indicated primers using Phanta Max Super-Fidelity DNA Polymerase (Vazyme). Then the PCR products were fused to the linearized vector through homologous recombination using the ClonExpress Ultra One Step Cloning Kit (Vazyme). Note that sequences encode the XTEN linker (SGSETPGTSESATPES), SV40 NLS (SPKKKRKVEAS), and HIV NES (LQLPPLERLTL) are added to the 5' end of the primers.

For the dCasRx(C)-NES-M3 and -M3^{D395A} constructs, the RNA m^{6}A methyltransferase domain of METTL3 (M3) was cloned from the cDNA of HEK293T cells. The methyltransferase-impaired M3^{D395A} was generated with the Mut Express II Fast Mutagenesis Kit V2 (Vazyme). Then the M3 or M3^{D395A} was fused to the C terminus of the FKBP-dCasRx(C) fragment through homologous recombination using the ClonExpress Ultra One Step Cloning Kit (Vazyme).

The CasRx gRNAs were golden-gate cloned into the pXR003-CasRx-gRNA (Addgene plasmid no. 109053) with BbsI restriction enzyme (NEB) and T4 DNA ligase (NEB), which had constitutive gRNA expression driven by the U6 promoter. PspCas13b gRNAs were golden-gate cloned into the pC0043-PspCas13b crRNA backbone (Addgene plasmid no. 103854) with BbsI enzyme (NEB) and T4 DNA ligase (NEB). We amplified these plasmids using the TStbl3 (TSINGKE) competent cells.

The CasRx (Addgene plasmid no. 109049), dCasRx (Addgene plasmid no. 109050), CasRx gRNA cloning backbone (Addgene plasmid no. 109053), PspCas13b (Addgene plasmid no. 103862), and PspCas13b gRNA cloning backbone (Addgene plasmid no. 103854) were purchased from Addgene.

Guideline to determine the split site of Cas13 protein

First, because the members of Cas13 family have two HEPN domains which are both indispensable for RNA cleavage (Makarova et al., 2020), so we suggest choosing sites between two HEPN domains that allow for separation of two catalytic domains into N- and C- fragments. Second, we recommend referring to the determined crystal structure of the Cas13 protein, or predicting the structure of the Cas13 protein by AlphaFold if the structure has not been determined yet. This will allow for selecting split sites from the structure or non-structure region. Third, we suggest to choose split sites in both the structure and non-structure regions.

gRNA design

The cas13design (https://cas13design.nygenome.org) is a flexible tool that is specifically developed to design optimal gRNAs with robust targeting efficiency for CasRx (Wessels et al., 2020). Thus, we applied cas13design to design optimal gRNAs for CasRx-mediated knockdown of *EGFR*, *VHL*, and *PNPLA2* mRNAs. To target *KRAS* mRNAs, gRNA used in the previous work by Abudayyeh et al. was adopted (Abudayyeh et al., 2017). To target *GAPDH* mRNAs, the gRNA used in the previous work by Wilson et al. was adopted (Wilson et al., 2020). All the gRNA spacer sequences were available in Supplementary Table S2.

Supplementary materials

Cell Culture

HEK293T cells were cultured with Dulbecco's Modification of Eagle's Medium (Corning) supplemented with 10% fetal bovine serum (Lonza). Plasmocin Prophylactic (Invivogen) was added to the culture medium at a concentration of 5 μg/ml to prevent mycoplasma contamination. Cells were cultured at 37 °C and 5% CO_2 in a humidified atmosphere.

Transfection

HEK293T cells were cultured and passaged in the 6-well plate until they reached approximately 80% confluency. Cells were co-transfected with the indicated Cas13 split constructs and the corresponding gRNA plasmid at a mass ratio of 2:1 using Lipofectamine 3000 (Thermo) according to the manufacturer's instructions. DMSO or rapamycin (200 nM) was added to the culture medium at 24 h after the transfection. After 48 h of rapamycin induction, cells were washed with PBS and followed by subsequent experiments.

RIP-qPCR

HEK293T cells were first transfected with the split-dCasRx conjugated with M3 or $M3^{D395A}$ plasmids for 24 h, then DMSO or rapamycin (200 nM) was added to the culture medium and maintained for 48 h. Next, the total RNA was extracted using TRIzol reagent (Invitrogen). Then 50 μg total RNA was fragmented to 200 nt at 94°C for 4 min using the Magnesium RNA Fragmentation Module (NEB E6150S). The RNA fragments were purified and concentrated by ethanol precipitation. One-tenth of the fragmented RNA was saved as input. Then, the fragmented RNA was incubated with 2 μg m^6A antibody (SYSY, 202003) or IgG (Beyotime A7028) and 5 μl RNase Inhibitor (Beyotime R0102) in 1 ml RIP buffer (10 mM Tris-HCl pH 7.4, 150 mM NaCl, and 0.1% NP-40) at 4 °C for shaking overnight. 40 μl protein A/G beads (MCE, HY-K0202) were washed twice with RIP buffer, and then incubated in RNA-RIP buffer mix and RNase Inhibitor (Beyotime R0102) at 4 °C for 4 h. RNA fragments containing m^6A on the beads was eluted with 200 μl IPP buffer containing 6.7 mM N^6-methyladenosine (MCE) and 5 μl RNase Inhibitor (Beyotime R0102), and then purified by RNA clean & concentrator kit (ZYMO), as IP. The m^6A level of certain transcripts was subsequently analyzed by RT-qPCR.

RNA isolation and RT-qPCR

Total RNA was isolated using TRIzol Reagent (Invitrogen), and complementary DNA was generated by HiScript® III All-in-one RT SuperMix (Vazyme). RT-qPCR was performed using the ChamQ Universal SYBR qPCR Master Mix (Vazyme) on the CFX96 Real-Time PCR System (Bio-Rad). The housekeeping gene 18S RNA was used as the internal control. Primers used for RT-qPCR are listed in Supplementary Table S1.

Immunofluorescence staining

HEK293T cells were transfected with sCasRx plasmids, followed by DMSO or rapamycin (200 nM) treatment after 24 h. Cells were passaged and cultured on coverslips for 24 h and then fixed with 4% paraformaldehyde for 10 min at room temperature, followed by methanol treatment for 10 min at -20°C. The fixed cells were washed with IF buffer (PBS supplemented with 0.2% TritonX) three times, blocked with 5% BSA in IF buffer for 30 min, and then incubated with rabbit anti-Flag (Sigma, F7425) or mouse anti-HA (Sigma, F3663) antibody for 2 h at room temperature. After washing with IF buffer three times, cells were then incubated with anti-rabbit (Abcam, Alexa Fluor® 488, ab150077) or anti-mouse secondary antibody (Abcam, Alexa Fluor® 647, ab150115) for 40 min at

room temperature, stained with DAPI (Abcam, ab228549) for 10 min and washed again with IF buffer for three times. Coverslips were finally mounted onto slides with ProLong Gold antifade reagent (Invitrogen) and images were acquired using a confocal microscope (OLYMPUS IX83-FV3000-OSR).

RNA sequencing and analysis to evaluate off-target effects of the sCasRx system

HEK293T cells were transfected with sCasRx.v2 plasmids expressing CasRx(N), CasRx(C) and *EGFR* or non-targeting gRNA, the rapamycin (200 nM) was added to the culture medium at 24 h after the transfection. After 48 h of rapamycin induction, cells were washed with PBS and followed by subsequent experiments. For *EGFR* knockdown by the intact CasRx, the plasmids expressing CasRx and *EGFR* or non-targeting gRNA were transfected to HEK293T cells and cells were harvested at 48 h after transfection. Total RNA was extracted following the protocol of TRIzol reagent (Invitrogen). All RNA-Seq libraries were constructed using the NEBNext Ultra Directional RNA Library Prep Kit for Illumina (NEB) according to the manufacturer’s instructions. The library quality was assessed using Agilent 4200 Bioanalyzer, which includes the product size and concentration. Then, RNA-seq libraries were subjected to paired-end 150-bp (PE150) sequencing on the Illumina NovaSeq 6000 platform (Illumina). Raw reads were filtered by removing adapters and low-quality reads with the fastp (v.0.23.2). The filtered RNA-seq reads of RNA knockdown experiments were aligned to the hg38 reference genome with HISAT2 (v.2.2.1). All uniquely mapped reads were processed by featureCount (v.2.0.3) to generate a read count matrix. DESeq2 (v.1.38.3) was used to calculate differentially expressed genes. Genes with |log2 (fold change) |>1 and *P* value < 0.01 were classified as differentially expressed genes. A customized python script was used to calculate fragments per kilobase per million mapped fragments (FPKM) values from the read count matrix for plotting visualization.

Protein structure prediction

Prediction runs were executed using AlphaFold (v2.2.0) and a modified version of ColabFold (v1.5.0) which was available at github (https://github.com/sokrypton/ColabFold). The max_release_date parameter was set to 10-05-2022 when the simulations were run such that template information was used for structure modeling. For all targets, five models were generated and all presets were kept the same.

Statistical analysis

P values were calculated using unpaired two-sided Student’s *t*-tests on GraphPad Prism 9. **P* < 0.05, ***P* < 0.01, ****P* < 0.001. n.s. stands for not significant. The values represent technical replicates, and all the experiments are replicated at least three independent times. Error bars represent mean ± s.d.

References

Abudayyeh, O.O., Gootenberg, J.S., Essletzbichler, P., Han, S., Joung, J., Belanto, J.J., Verdine, V., Cox, D.B.T., Kellner, M.J., Regev, A., *et al.* (2017). RNA targeting with CRISPR-Cas13. Nature *550*, 280-284.
Makarova, K.S., Wolf, Y.I., Iranzo, J., Shmakov, S.A., Alkhnbashi, O.S., Brouns, S.J.J., Charpentier, E., Cheng, D., Haft, D.H., Horvath, P., *et al.* (2020). Evolutionary classification of CRISPR-Cas systems: a burst of class 2 and derived variants. Nat Rev Microbiol *18*, 67-83.
Wessels, H.H., Mendez-Mancilla, A., Guo, X., Legut, M., Daniloski, Z., and Sanjana, N.E. (2020). Massively parallel Cas13 screens reveal principles for guide RNA design. Nat Biotechnol *38*, 722-727.
Wilson, C., Chen, P.J., Miao, Z., and Liu, D.R. (2020). Programmable m(6)A modification of cellular RNAs with a Cas13-directed methyltransferase. Nat Biotechnol *38*, 1431-1440.

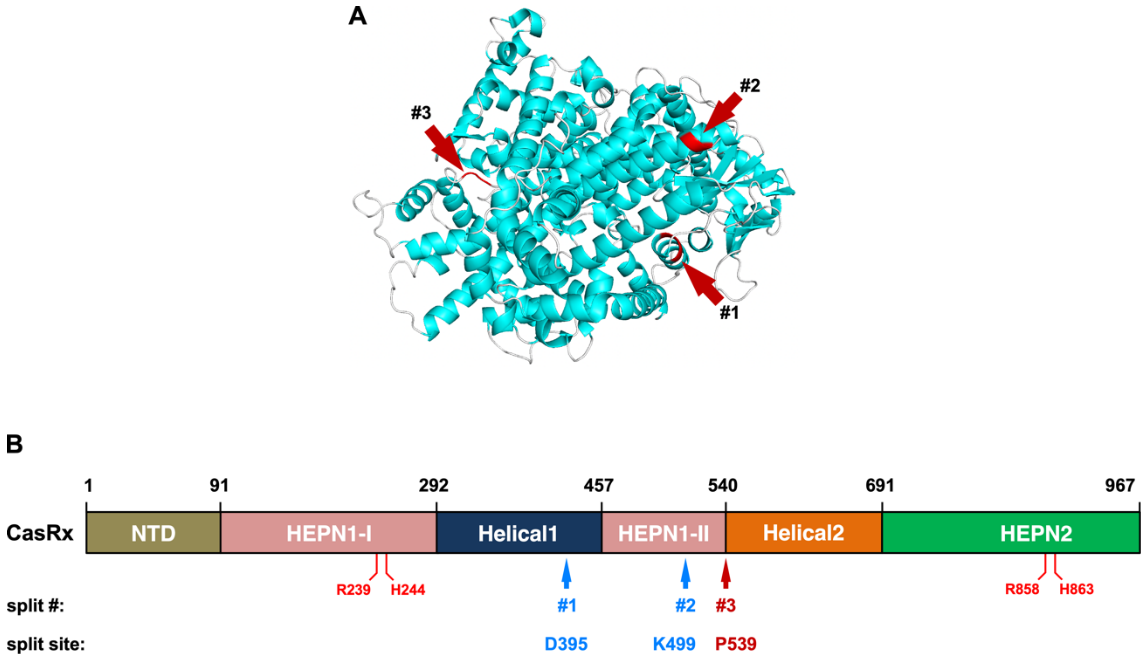

Supplementary Figure S1. The predicted structure and split sites of the CasRx protein. (**A**) The predicted structure of the CasRx protein by AlphaFold2. Red arrows indicate three split sites. (**B**) Three split sites were determined by combining the protein structure and domain organization of the CasRx protein. Two blue arrows marked the number 1 and 2 split sites located in the structured region, while the red arrow marked the number 3 split site located in the unstructured region.

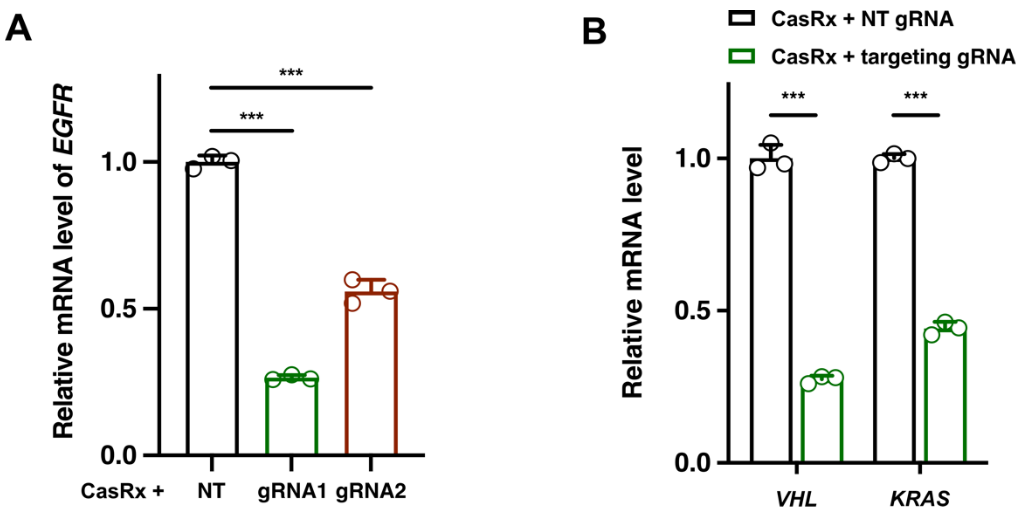

Supplementary Figure S2. The targeting efficiency of CasRx gRNAs. (**A**) The targeting efficiency of *EGFR* gRNAs. (**B**) The targeting efficiency of *VHL* and *KRAS* gRNAs. RNA levels were determined by RT-qPCR. NT stands for non-targeting gRNA. Values and error bars represent mean±s.d., ****P* < 0.001.

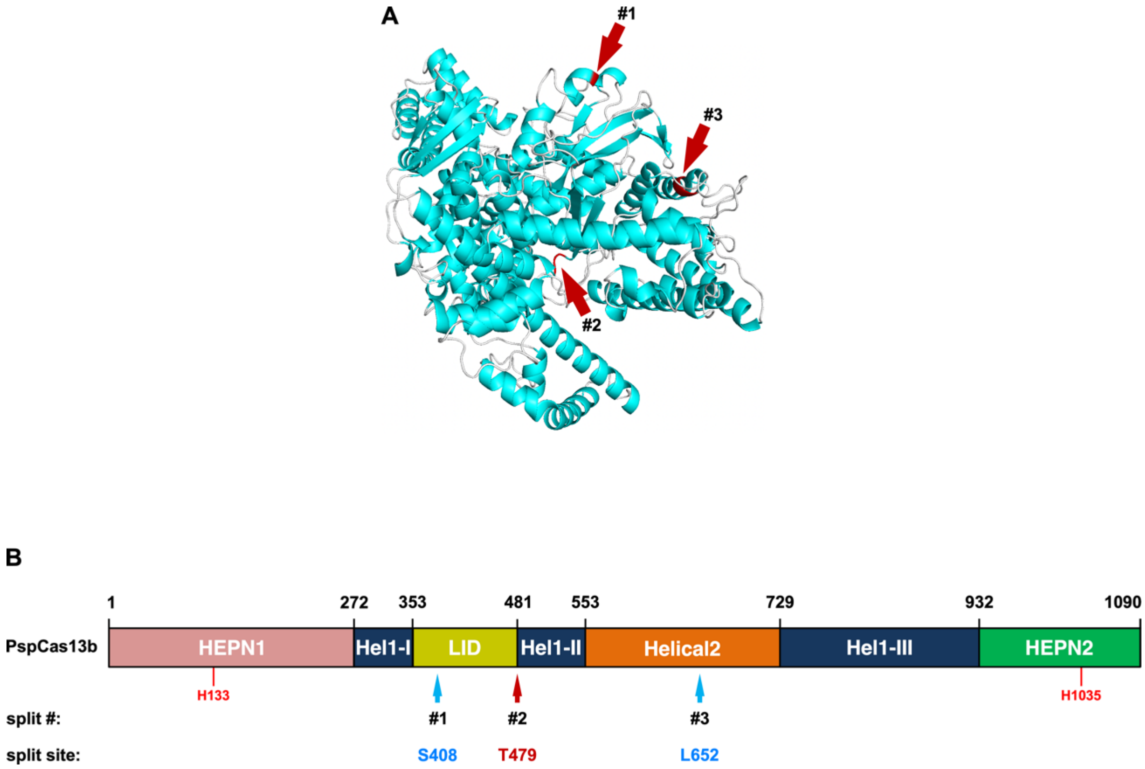

Supplementary Figure S3. The predicted structure and split sites of the PspCas13b protein. (**A**) The predicted structure of the PspCas13b protein by AlphaFold2. Red arrows indicate three split sites. (**B**) Three split sites were determined by combining the protein structure and domain organization of the PspCas13b protein. Two blue arrows marked number 1 and 3 split sites located in the structured region, while the red arrow marked the number 2 split site located in the unstructured region.

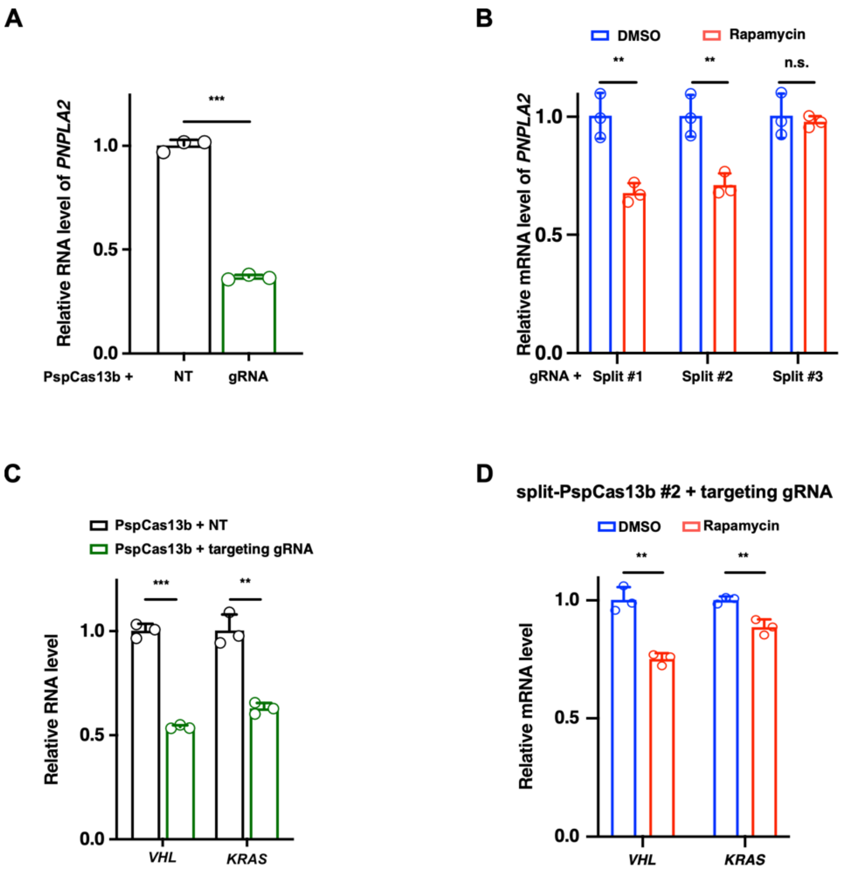

Supplementary Figure S4. Validation of the inducible split-Cas13b system. (**A**) The targeting efficiency of *PNPLA2* gRNA in the CRISPR-PspCas13b system. (**B**) The rapamycin-induced knockdown efficiency of split-PspCas13b on *PNPLA2* mRNAs. (**C**) The targeting efficiency of *VHL* and *KRAS* gRNAs in the CRISPR-PspCas13b system. (**D**) The rapamycin-induced knockdown efficiency of split-PspCasR13b on *VHL* and *KRAS* mRNAs. RNA levels were determined by RT-qPCR. NT stands for non-targeting gRNA. Values and error bars represent mean±s.d., **P < 0.01, ***P < 0.001, n.s. stands for not significant.

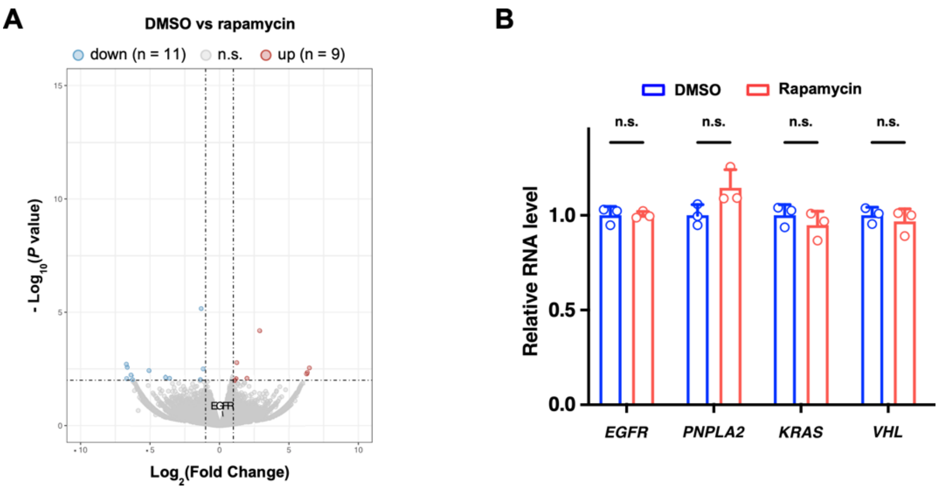

Supplementary Figure S5. Rapamycin treatment shows no obvious effect on target RNAs. (**A**) Differential gene expression analysis of RNA-seq of DMSO or rapamycin treatment in HEK293T cells (two biological replicates). (**B**) The mRNA level of target genes was examined by RT-qPCR. Values and error bars represent mean±s.d., n.s. stands for not significant.

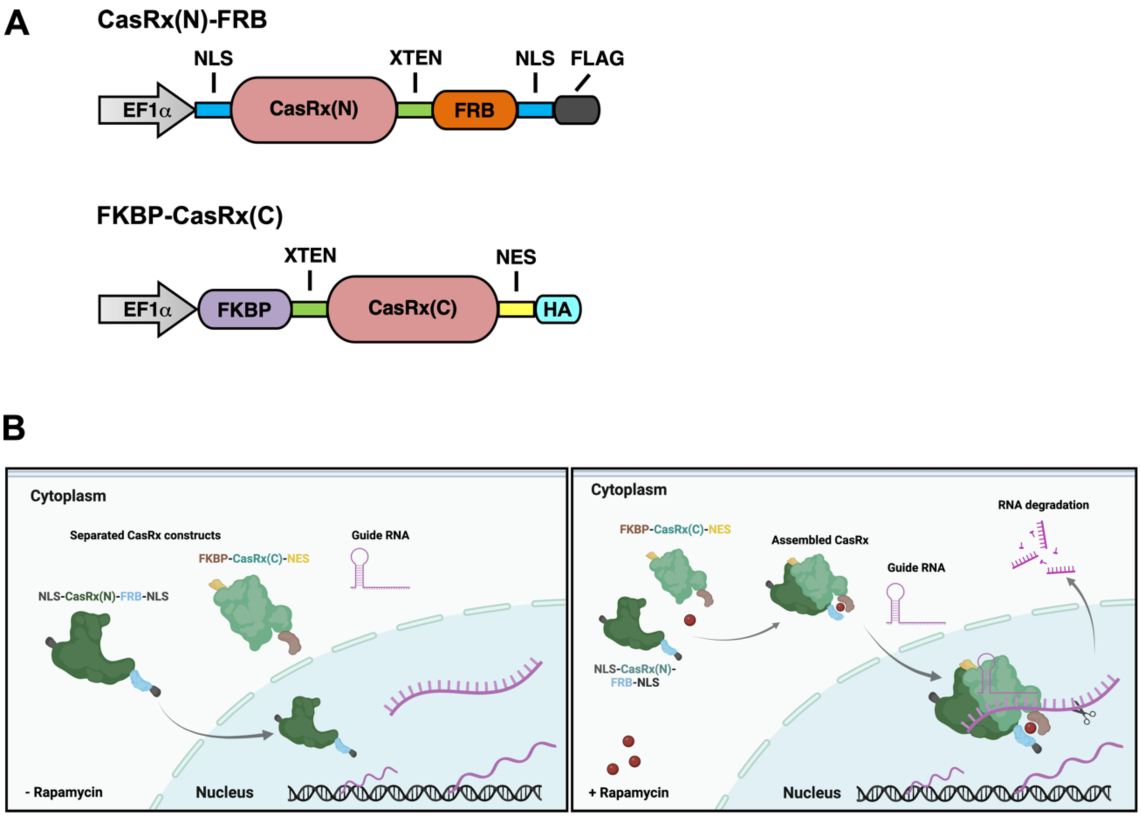

Supplementary Figure S6. Optimization of the split-CasRx system. (**A**) Optimized construction of NLS-CasRx(N)-FRB-NLS and FKBP-CasRx(C)-NES fusions of sCasRx.v2. (**B**) Proposed working model for the optimized sCasRx.v2 in the absence (left panel) or presence (right panel) of rapamycin.

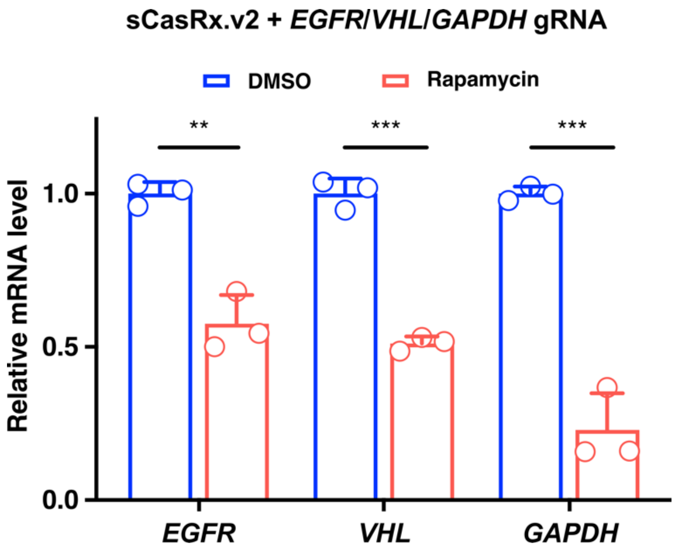

Supplementary Figure S7. Inducible knockdown of multiple mRNAs by sCasRx.v2. The mRNA level of *EGFR*, *VHL*, and *GAPDH* was determined by RT-qPCR. **$P < 0.01$, ***$P < 0.001$.

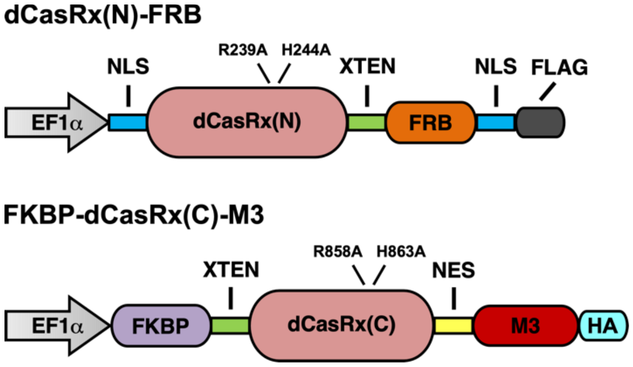

Supplementary Figure S8. Construction of the inducible RNA m^{6}A editing tool by conjugating split-dCasRx and RNA m^{6}A methyltransferase domain of METTL3 (M3).

Supplementary materials

Supplementary Table S1. Primers used in this work

Name	Sequences (5'-3')	Usage	Source
q*EGFR*-F	GTGCGGAAGAGAAAGAATACCA	RT-qPCR	Figure 1, S2, S5, and S7
q*EGFR*-R	CCAAAGGTCATCAACTCCCA	RT-qPCR	Figure 1, S2, S5, and S7
q*VHL*-F	GACGGACAGCCTATTTTTGCC	RT-qPCR	Figure 1, S2, S4, and S5
q*VHL*-R	TCTGCACATTTGGGTGGTCTT	RT-qPCR	Figure 1, S2, S4, and S5
q*KRAS*-F	GAAACATCAGCAAAGACAAGAC	RT-qPCR	Figure 1, S2, S4, and S5
q*KRAS*-R	ATAGAAGGCATCATCAACACC	RT-qPCR	Figure 1, S2, S4, and S5
q*PNPLA2*-F	AAGATCATCCGCAGTTTCCT	RT-qPCR	Figure S4 and S5
q*PNPLA2*-R	TTGTCTGAAATGCCACCATCC	RT-qPCR	Figure S4 and S5
q18S RNA-F	GGATGTAAAGGATGGAAAATACA	RT-qPCR	Figure 1, S4, and S5
q18S RNA-R	TCCAGGTCTTCACGGAGCTTGTT	RT-qPCR	Figure 1, S4, and S5
qGAPDH-F	CATCACTGCCACCCAGAAGA	m^6A RT-qPCR	Figure 1 and S7
qGAPDH-R	CAGTAGAGGCAGGGATGATGTT	m^6A RT-qPCR	Figure 1 and S7
cPXR001-F	GGACCTAAGAAAAAGAGGAAGG	Linearizing the PXR001 vector	
cPXR001-R	CTTCTTGGGGCTCATGGTGGCCGTACGTCACGACAC	Linearizing the PXR001 vector	
cRx(N)-F	ATGAGCCCCAAGAAGAAGAGAAAGGTGGAGGCCAGCATCGAAAAAAAAAAGTCCTT	Cloning CasRx(N)#1, #2, and #3 (with NLS in the 5')	
cRx(N)#1-R	CTCGCTTGTTCCTGGTGTCTCGCTGCCAGAAGCGGGCAGGATTCTGGGCA	Cloning CasRx(N)#1 (with XTEN in the 5')	
cRx(N)#2-R	CTCGCTTGTTCCTGGTGTCTCGCTGCCAGACTTCAGGAAGCTCTGGATGTTATCG	Cloning CasRx(N)#2 (with XTEN in the 5')	
cRx(N)#3-R	CTCGCTTGTTCCTGGTGTCTCGCTGCCAGAAGGTTCTCCCATTCTAGCGAAGG	Cloning CasRx(N)#3 (with XTEN in the 5')	
cFRB-F	AGGCCTTCATGCCACATCTCGCTCTCTGGTGTTGCTGACTCGCTTGTTCCTGGTGTCT	Cloning FRB (with XTEN in the 5')	
cFRB-R	CTGCTTTGAGATTCGTCGGAACAC	Cloning FRB (with NLS in the 5')	
cRx(C)#1-F	AGACACCAGGAACAAGCGAGTCAGCAACACCAGAGAGCGGCCGTGATGTTTCCGCCTT	Cloning CasRx(C)#1 (with XTEN in the 5')	
cRx(C)#2-F	AGACACCAGGAACAAGCGAGTCAGCAACACCAGAGAGCGTGATGCCTCTCATCGGAGTC	Cloning CasRx(C)#2 (with XTEN in the 5')	
cRx(C)#3-F	AGACACCAGGAACAAGCGAGTCAGCAACACCAGAGAGCATTGCCGATGCCAGGAGGGC	Cloning CasRx(C)#3 (with XTEN in the 5')	
cRx(C)-R	CTTTTTCTTAGGTCCGGATCCGGAATTGCCGGACACCTTCTTTTTC	Cloning CasRx(C) #1, #2, and #3 (with NLS in the 5')	
cFKBP-F	GGAGTGCAGGTGGAAACCATCTC	Cloning FKBP	
cFKBP-R	CTCGCTTGTTCCTGGTGTCTCGCTGCCAGATTCCAGTTTTAGAAGCTCCACATCGAAG	Cloning FKBP (with XTEN in the 5')	
c*M3*-F	CTGCCTCCACTTGAAAGACTGACACTGCAAGAATTCTGTGACTATGGAACCAAGG	Cloning M3 (with NES in the 5')	
c*M3*-R	AGAAGTTTGTTGCGCCGGATCCAGCGTAATCTGGAACATCGTATGGGTATAAATTCTTAGGTTTAGAGATGATACC	Cloning M3 (with HA in the 5')	
M3-mutF	GATGGCTgcgCCACCCTGGGATATTCACATGGA	$M3^{D395A}$ mutagenesis	
M3-mutR	AGGGTGGcgcAGCCATCACAACTGCAAACTTGC	$M3^{D395A}$ mutagenesis	

	AGACACCAGGAACAAGCGAGTCAGCAACACCAGAGAG CATTGCCGATGCCAGGAGGGC	Cloning dCasRx(C)#3 (with XTEN in the 5’)
dCasRx(C)#3-R	AGTCTTTCAAGTGGAGGCAGTTGAAGGGAATTGCCGGA CACCTTCTTTTTC	Cloning dCasRx(C)#3 (with NES in the 5’)

Supplementary Table S2. Guide RNA spacer sequences used in this study

**Name**	**Sequences (5’-3’)**	**Usage**	**Source**
NT gRNA	TCACCAGAAGCGTACCATACTCG	Control	Figure 1, S2, and S4
EGFR gRNA1	CACTGCTTTGTGGCGCGACCCTT	CasRx-mediated RNA targeting	Figure 1 and S2
EGFR gRNA2	CTATCCTCCGTGGTCATGCTCC	CasRx-mediated RNA targeting	Figure 1 and S2
VHL gRNA	CAACAAAAATAGAGGGCAGAACCT	CasRx-mediated RNA targeting	Figure S2
KRAS gRNA	ACCATAGGTACATCTTCAGAGTCCTTAA	CasRx-mediated RNA targeting	Figure S2
PNPLA2 gRNA	AGGCAGACCCCGGTGACCAGCGCCGTGGCC	PspCas13b-mediated RNA targeting	Figure S4
VHL gRNA	GTGTATCTAATAGGGATCTTTTTTCTTCCT	PspCas13b-mediated RNA targeting	Figure S4
KRAS gRNA	GGACCATAGGTACATCTTCAGAGTCCTTAA	PspCas13b-mediated RNA targeting	Figure S4
NT gRNA	GTAATGCCTGGCTTGTCGACGCATAGTCTG	Control	Figure 1 and S7
GAPDH gRNA	AGCCCCGCGGCCATCACGCCACAGTTTCCC	Split-dCasRx-M3-mediated m^6A editing	Figure 1 and S7

Supplementary Sequences 1. Amino acid sequences of sCasRx.v1 fusions.

NLS-CasRx(N)#3-XTEN-FRB-NLS-FLAG

MSPKKKRKVEASIEKKKSFAKGMGVKSTLVSGSKVYMTTFAEGSDARLEKIVEGDSIRSVNEGEAFSA
EMADKNAGYKIGNAKFSHPKGYAVVANNPLYTGPVQQDMLGLKETLEKRYFGESADGNDNICIQVIHN
ILDIEKILAEYITNAAYAVNNISGLDKDIIGFGKFSTVYTYDEFKDPEHHRAAFNNNDKLINAIKAQYDEFD
NFLDNPRLGYFGQAFFSKEGRNYIINYGNECYDILALLSGLRHWVVHNNEEESRISRTWLYNLDKNLD
NEYISTLNYLYDRITNELTNSFSKNSAANVNYIAETLGINPAEFAEQYFRFSIMKEQKNLGFNITKLREVM
LDRKDMSEIRKNHKVFDSIRTKVYTMMDFVIYRYYIEEDAKVAAANKSLPDNEKSLSEKDIFVINLRGSF
NDDQKDALYYDEANRIWRKLENIMHNIKEFRGNKTREYKKKDAPRLPRILPAGRDVSAFSKLMYALTM
FLDGKEINDLLTTLINKFDNIQSFLKVMPLIGVNAKFVEEYAFFKDSAKIADELRLIKSFARMGEPSGSET
PGTSESATPESEMWHEGLEEASRLYFGERNVKGMFEVLEPLHAMMERGPQTLKETSFNQAYGRDLM
EAQEWCRKYMKSGNVKDLTQAWDLYYHVFRRISKQSPKKKRKVEASDYKDDDDK

NLS-FKBP-XTEN-CasRx(C)#3- NLS-HA

MSPKKKRKVEASGVQVETISPGDGRTFPKRGQTCVVHYTGMLEDGKKFDSSRDRNKPFKFMLGKQE
VIRGWEEGVAQMSVGQRAKLTISPDYAYGATGHPGIIPPHATLVFDVELLKLESGSETPGTSESATPES
IADARRAMYIDAIRILGTNLSYDELKALADTFSLDENGNKLKKGKHGMRNFIINNVISNKRFHYLIRYGDP
AHLHEIAKNEAVVKFVLGRIADIQKKQGQNGKNQIDRYYETCIGKDKGKSVSEKVDALTKIITGMNYDQF
DKKRSVIEDTGRENAEREKFKKIISLYLTVIYHILKNIVNINARYVIGFHCVERDAQLYKEKGYDINLKKLE
EKGFSSVTKLCAGIDETAPDKRKDVEKEMAERAKESIDSLESANPKLYANYIKYSDEKKAEEFTRQINR
EKAKTALNAYLRNTKWNVIIREDLLRIDNKTCTLFRNKAVHLEVARYVHAYINDIAEVNSYFQLYHYIMQ
RIIMNERYEKSSGKVSEYFDAVNDEKKYNDRLLKLLCVPFGYCIPRFKNLSIEALFDRNEAAKFDKEKKK
VSGNSSPKKKRKVEASYPYDVPDYA

Supplementary Sequences 2. Amino acid sequences of sCasRx.v2 fusions.

NLS-CasRx(N)#3-XTEN-FRB-NLS-FLAG

Same as the correlated one with sCasRx.v1

FKBP12-XTEN-CasRx(C)#3-NES-HA

MGVQVETISPGDGRTFPKRGQTCVVHYTGMLEDGKKFDSSRDRNKPFKFMLGKQEVIRGWEEGVAQ
MSVGQRAKLTISPDYAYGATGHPGIIPPHATLVFDVELLKLESGSETPGTSESATPESIADARRAMYIDA
IRILGTNLSYDELKALADTFSLDENGNKLKKGKHGMRNFIINNVISNKRFHYLIRYGDPAHLHEIAKNEAV
VKFVLGRIADIQKKQGQNGKNQIDRYYETCIGKDKGKSVSEKVDALTKIITGMNYDQFDKKRSVIEDTG
RENAEREKFKKIISLYLTVIYHILKNIVNINARYVIGFHCVERDAQLYKEKGYDINLKKLEEKGFSSVTKLC
AGIDETAPDKRKDVEKEMAERAKESIDSLESANPKLYANYIKYSDEKKAEEFTRQINREKAKTALNAYL
RNTKWNVIIREDLLRIDNKTCTLFRNKAVHLEVARYVHAYINDIAEVNSYFQLYHYIMQRIIMNERYEKSS
GKVSEYFDAVNDEKKYNDRLLKLLCVPFGYCIPRFKNLSIEALFDRNEAAKFDKEKKKVSGNSLQLPPL
ERLTLYPYDVPDYA

Supplementary Sequences 3. Amino acid sequences of split-dCasRx-M3/M3^{D395A} fusions.

NLS-dCasRx(N)#3-XTEN-FRB-NLS-FLAG

MSPKKKRKVEASIEKKKSFAKGMGVKSTLVSGSKVYMTTFAEGSDARLEKIVEGDSIRSVNEGEAFSA
EMADKNAGYKIGNAKFSHPKGYAVVANNPLYTGPVQQDMLGLKETLEKRYFGESADGNDNICIQVIHN
ILDIEKILAEYITNAAYAVNNISGLDKDIIGFGKFSTVYTYDEFKDPEHHRAAFNNNDKLINAIKAQYDEFD
NFLDNPRLGYFGQAFFSKEGRNYIINYGNECYDILALLSGLAHWVVANNEEESRISRTWLYNLDKNLDN
EYISTLNYLYDRITNELTNSFSKNSAANVNYIAETLGINPAEFAEQYFRFSIMKEQKNLGFNITKLREVML
DRKDMSEIRKNHKVFDSIRTKVYTMMDFVIYRYYIEEDAKVAAANKSLPDNEKSLSEKDIFVINLRGSFN
DDQKDALYYDEANRIWRKLENIMHNIKEFRGNKTREYKKKDAPRLPRILPAGRDVSAFSKLMYALTMFL
DGKEINDLLTTLINKFDNIQSFLKVMPLIGVNAKFVEEYAFFKDSAKIADELRLIKSFARMGEPSGSETPG
TSESATPESEMWHEGLEEASRLYFGERNVKGMFEVLEPLHAMMERGPQTLKETSFNQAYGRDLMEA
QEWCRKYMKSGNVKDLTQAWDLYYHVFRRISKQSPKKKRKVEASDYKDDDDK

FKBP12-XTEN-dCasRx(C)#3-NES-M3-HA

MGVQVETISPGDGRTFPKRGQTCVVHYTGMLEDGKKFDSSRDRNKPFKFMLGKQEVIRGWEEGVAQ
MSVGQRAKLTISPDYAYGATGHPGIIPPHATLVFDVELLKLESGSETPGTSESATPESIADARRAMYIDA
IRILGTNLSYDELKALADTFSLDENGNKLKKGKHGMRNFIINNVISNKRFHYLIRYGDPAHLHEIAKNEAV
VKFVLGRIADIQKKQGQNGKNQIDRYYETCIGKDKGKSVSEKVDALTKIITGMNYDQFDKKRSVIEDTG
RENAEREKFKKIISLYLTVIYHILKNIVNINARYVIGFHCVERDAQLYKEKGYDINLKKLEEKGFSSVTKLC
AGIDETAPDKRKDVEKEMAERAKESIDSLESANPKLYANYIKYSDEKKAEEFTRQINREKAKTALNAYL
RNTKWNVIIREDLLRIDNKTCTLFANKAVALEVARYVHAYINDIAEVNSYFQLYHYIMQRIIMNERYEKSS
GKVSEYFDAVNDEKKYNDRLLKLLCVPFGYCIPRFKNLSIEALFDRNEAAKFDKEKKKVSGNSLQLPPL
ERLTLQEFCDYGTKEECMKASDADRPCRKLHFRRIINKHTDESLGDCSFLNTCFHMDTCKYVHYEIDA
CMDSEAPGSKDHTPSQELALTQSVGGDSSADRLFPPQWICCDIRYLDVSILGKFAVVMADPPWDIHM
ELPYGTLTDDEMRRLNIPVLQDDGFLFLWVTGRAMELGRECLNLWGYERVDEIIWVKTNQLQRIIRTG
RTGHWLNHGKEHCLVGVKGNPQGFNQGLDCDVIVAEVRSTSHKPDEIYGMIERLSPGTRKIELFGRP
HNVQPNWITLGNQLDGIHLLDPDVVARFKQRYPDGIISKPKNLYPYDVPDYA

FKBP12-XTEN-dCasRx(C)#3-NES-M3^{D395A}-HA

MGVQVETISPGDGRTFPKRGQTCVVHYTGMLEDGKKFDSSRDRNKPFKFMLGKQEVIRGWEEGVAQ
MSVGQRAKLTISPDYAYGATGHPGIIPPHATLVFDVELLKLESGSETPGTSESATPESIADARRAMYIDA
IRILGTNLSYDELKALADTFSLDENGNKLKKGKHGMRNFIINNVISNKRFHYLIRYGDPAHLHEIAKNEAV
VKFVLGRIADIQKKQGQNGKNQIDRYYETCIGKDKGKSVSEKVDALTKIITGMNYDQFDKKRSVIEDTG
RENAEREKFKKIISLYLTVIYHILKNIVNINARYVIGFHCVERDAQLYKEKGYDINLKKLEEKGFSSVTKLC
AGIDETAPDKRKDVEKEMAERAKESIDSLESANPKLYANYIKYSDEKKAEEFTRQINREKAKTALNAYL
RNTKWNVIIREDLLRIDNKTCTLFANKAVALEVARYVHAYINDIAEVNSYFQLYHYIMQRIIMNERYEKSS
GKVSEYFDAVNDEKKYNDRLLKLLCVPFGYCIPRFKNLSIEALFDRNEAAKFDKEKKKVSGNSLQLPPL
ERLTLQEFCDYGTKEECMKASDADRPCRKLHFRRIINKHTDESLGDCSFLNTCFHMDTCKYVHYEIDA
CMDSEAPGSKDHTPSQELALTQSVGGDSSADRLFPPQWICCDIRYLDVSILGKFAVVMAAPPWDIHM
ELPYGTLTDDEMRRLNIPVLQDDGFLFLWVTGRAMELGRECLNLWGYERVDEIIWVKTNQLQRIIRTG
RTGHWLNHGKEHCLVGVKGNPQGFNQGLDCDVIVAEVRSTSHKPDEIYGMIERLSPGTRKIELFGRP
HNVQPNWITLGNQLDGIHLLDPDVVARFKQRYPDGIISKPKNLYPYDVPDYA
